# Supplementary material for: Complexome profiling on the Chlamydomonas lpa2 mutant reveals insights into PSII biogenesis and new PSII associated proteins
Source: J Exp Bot. 2021 Aug 26;73(1):245–62. doi: 10.1093/jxb/erab390 (PMC8730698; doi:10.1093/jxb/erab390)
Supplement: erab390_suppl_Supplementary_Dataset_S1 [file erab390_suppl_supplementary_dataset_s1.zip › Supplemental Dataset 1 - Excel List and all profiles/plots/OTA1_Cre11.g474800.html]

Trivial name: OTA1  
  
Euclidean distance: 9525.68  
Mean Intensity (WT): 1207.90  
Mean Intensity (Mut): 1091.13  
Distance: 7.89  
  
MapMan: tetrapyrrole synthesis.GSA;amino acid metabolism.degradation.glutamate family.arginine;amino acid metabolism.synthesis.glutamate family.proline.ornithine aminotransferase  
  
p value of intensity sums Welch test: 0.8652
